# Measuring Interests Not Minutes: Development and Validation of the Adolescents’ Digital Technology Interactions and Importance Scale (ADTI)

**DOI:** 10.2196/16736

**Published:** 2020-02-12

**Authors:** Megan A Moreno, Kole Binger, Qianqian Zhao, Jens Eickhoff

**Affiliations:** 1 Department of Pediatrics University of Wisconsin-Madison Madison, WI United States; 2 Department of Biostatistics and Medical Informatics University of Wisconsin-Madison Madison, WI United States

**Keywords:** technology, adolescents, methodology, survey, social media, screen time, instrument development

## Abstract

**Background:**

Interactive digital technology use is integral to adolescents’ lives and has been associated with both health benefits and risks. Previous studies have largely focused on measuring the quantity of technology use or understanding the use of specific platforms. To better understand adolescents’ interactive digital technology use, we need new approaches that consider technology interactions and their importance.

**Objective:**

This study aimed to develop an assessment tool to evaluate adolescents’ digital technology interactions and their perceived importance.

**Methods:**

We used a validated scale development approach comprising 2 initial steps to create an item pool: item pool development and item pool refinement. These steps relied upon empirical literature review and an expert convening. We then evaluated the item pool using a Web-based survey. Data were collected via Qualtrics panel recruitment from a national sample of 12- to 18-year-olds. Participant data were randomly split into a development subsample for exploratory factor analysis (EFA) and a test subsample for confirmatory factor analysis (CFA). We assessed Cronbach alpha as well as model fit characteristics including root mean square error of approximation (RMSEA) and comparative fit index (CFI).

**Results:**

Our initial item pool had 71 items and the refined item pool contained 40. A total of 761 adolescents assessed the item pool via Web-based survey. Participants had a mean age of 14.8 (SD 1.7) years and were 52.8% (402/761) female and 77.5% (590/761) white. The EFA analysis included 500 participants and an 18-item draft scale was created. The CFA included 261 participants to test the draft scale. Adequate model fit for the scale was indicated by an RMSEA of 0.063 and a CFI of 0.95. The final scale included 18 items in a 3-factor model, with Cronbach alpha for the 3 factors of .87 (factor 1), .90 (factor 2) and .82 (factor 3). The 3 factors were named (1) technology to bridge online and offline experiences, (2) technology to go outside one’s identity or offline environment, and (3) technology for social connection.

**Conclusions:**

The resulting Adolescents’ Digital Technology Interactions and Importance (ADTI) scale is a promising and psychometrically validated tool for identifying the importance of distinct technology interactions. The scale is informed by relevant theory and expert input. The 3 subscales have utility for future studies to understand whether certain subscale score ranges are associated with health or well-being outcomes.

## Introduction

### Background

Adolescents today are often considered digital natives given they are growing up in an immersive technological society. The majority of adolescents have a personal smartphone and engage with digital media; approximately 45% of adolescents describe that they are online *almost constantly* [1]. These findings illustrate that technology use is nearly ubiquitous and highly important to today’s adolescents. Through previous research, our understanding of how these consistent technology interactions can impact adolescents’ health and well-being has grown. Studies illustrate ways in which technology interactions offer adolescents’ well-being benefits, including opportunities for content creation and social support [2]. However, digital technology use has also been associated with negative health outcomes including impaired sleep [3-5], decreased physical activity [4,6,7], problematic internet use [8-10], and risk for depression [11,12]. Little is known about the association between adolescents’ perceived importance of particular technology behaviors and benefits or risks for adolescents.

### Quantity of Technology Use

The vast majority of studies in this area have focused on technology assessments of quantity of time spent using technology. Designing research studies to assess quantity of technology use has 3 main challenges, one of which is that self-reporting the quantity of technology use is subject to recall bias. Previous studies have shown that reported amount of time spent on technology use is often inaccurate [13,14]. Second, technology use occurs across multiple platforms. During any given day, an adolescent may interact with a personal smartphone, a school tablet, and a home computer. This multidevice use creates measurement challenges for both self-report and passive sensing research methods. For self-report, remembering use across multiple spaces and devices may increase the likelihood of reporting errors. For passive sensing measures, such as applications that track media use, this multidevice use means that measuring only 1 device does not capture the full range of daily use. Although some commercially available applications have evolved to passively track media use across more than 1 device, these approaches can present ethical issues as well as compatibility issues with some operating systems. A final challenge is that norms and expectations of time spent on technology have evolved over the years, thus the definition of *too much time online* has not remained a static target.

### Quality of Technology Use

Beyond these challenges in understanding *how much* adolescents use technology, measuring the amount of technology use time does not enhance our understanding of *how* adolescents use technology. Increasingly, researchers and health care providers are emphasizing that the quality of technology use, beyond just quantity of use, may be important in understanding links between technology use and health outcomes. A previous study examined adolescents’ social media use and compared passive scrolling behaviors with active engagement with others [15]. They found that passive scrolling behaviors were associated with negative mood, but actively engaged social media use was not. This study illustrated that particular technology behaviors and interactions are critical to understanding how mood may be affected by technology use.

This shift in thinking about technology beyond quantity of use is further illustrated by changes in the American Academy of Pediatrics’ (AAP) policy recommendations [16]. In 2016, the AAP media policy changed its recommendations from *2 hours a day or less of media and technology use* to promoting a customizable Family Media Use plan that represented both technology use time and behaviors [17]. The Family Media Use plan allows families to create household rules and guidelines around both quality and quantity of technology use. This dramatic shift in policy even included recommendations for youth to consider the importance of high-quality media and interact with that media, such as coviewing movies or coplaying video games with parents.

### Importance of Technology Interactions and Experiences

A novel approach to consider in assessing adolescents’ technology use is understanding the *importance* of particular technology interactions or experiences. Technology interactions that are perceived as important to adolescents are likely the ones that they spend the most time and effort in engaging with on a regular basis. It is possible that assessing the importance of technology interactions may provide more information to guide the motivation behind technology use and inform interventions and messaging. Thus, importance may be a novel way to measure both quantity and quality of technology interactions.

Understanding the importance of adolescents’ technology interactions may be informed by 3 theoretical approaches. The first approach to consider is the Uses and Gratifications model [18,19]. This theory has been applied to understand ways that people seek out types of technology to achieve particular needs or gratifications that are important to that individual. Example constructs represented in that scale include that technology may offer *social interaction*, *information seeking*, or *entertainment*. The Uses and Gratifications theory has several associated scales linked to the types of technology use, each of these scales is designed for a specific device or topic area such as cell phones [20], social media [18,19], and use of the internet for political information [21,22].

A second theoretical approach to consider is the Facebook Influence Model (FIM) [23]. The FIM describes ways in which social media, such as Facebook, may be influential to adolescents’ ideas, moods, or experiences. Example items from this model include *social media as a way to learn about new acquaintances*, *social media to connect to businesses*, and *social media as a way to procrastinate chores or studying*. However, assessing technology importance with the FIM is limited by its focus on social media.

Third, technology Affordances has also been used in understanding the aspects of technology design that may be important to users [24-26]. Example Affordances include *social affordances*, such as the capacity to build a social network, or tag users to engage them. At present, no measurement tool to assess affordances of digital technology among adolescents exists.

### Study Purpose

These valuable theories and conceptual approaches have formed a foundation by which we can continue to evolve our understanding of adolescents’ interactive technology behavior. A current gap in the literature is a validated approach to measure technology interactions that are important to adolescents. This assessment approach would go beyond the limitations and inaccuracies of measuring technology time. Furthermore, this approach would allow researchers to understand the aspects of technology that are important to adolescents, and thus likely represent much of adolescents’ time, effort, and attention. Previous theory could guide important measurement constructs, such as *technology to connect to others*. However, no current instrument can capture technology behaviors and their importance across the multiple platforms, devices, and behaviors involved in adolescent interactive digital technology use. For this study, we focused on digital technologies that promote interactive use (ie, social media, interactive gaming, and virtual reality [VR]). Thus, the purpose of this study was to develop a scale to assess digital technology interactions and their importance. We determined that the ideal tool would have certain characteristics. These characteristics and their supporting rationale are as follows: (1) the scale would be rooted in previous evidence and theory across disciplines, such that it incorporated existing scientific knowledge and acknowledged conceptual models; (2) the scale would be platform agnostic, such that it did not focus on name brand platforms or specific technology tools that may be impermanent; (3) the scale would be usable across emerging technologies such as VR to reflect novel technologies; (4) the scale would focus on the importance of specific technology interactions, such that it could identify interactions that were more or less important to an individual; and (5) the scale would demonstrate strong psychometric validation.

## Methods

### Study Design

To achieve our study aims, we used a validated scale development approach [27]. The first 2 steps focused on item pool development followed by item pool refinement. The resulting item pool was then evaluated via a Web-based survey among a sample of adolescents. Survey data were randomly divided into developmental and test subsamples for analyses. This study was reviewed and approved by the Institutional Review Board at University of Wisconsin—Madison.

### Item Pool Development: Theory and Evidence Review

To develop an item pool, we used 2 approaches. First, we reviewed existing scientific literature and identified relevant theory that described motivations, functionality, or experiences with technology use. This literature search was conducted by 2 investigators and focused on identification of theory specifically related to adolescents and technology/media use. We reviewed the published empirical literature as well as several media/technology textbooks that were cited within the empirical literature. The following databases were incorporated into our search: PubMed, CINAHL, PsychInfo, and Web of Science. Selected search keywords included “adolescent,” “media,” “technology,” “social media,” “theory,” “assessment,” and “measurement.” Following this search, we also consulted with 2 additional technology researchers outside our institution to review our search process and findings to ensure we had not missed relevant theory.

The result of this initial literature search was the identification of 3 key frameworks relevant to this study. These frameworks included Uses and Gratifications [28], the FIM [23], and the Affordances approach [29]. We then conducted a second literature search focused on these 3 conceptual approaches; we reviewed the scientific literature to identify any existing measurement scales tied to those approaches. The literature search included PubMed, CINAHL, PsychInfo, and Web of Science. Keywords included in the search consisted of the names and words within names of each of these 3 conceptual/theoretical models.

These existing scales were reviewed, and relevant survey items were added to the item pool. We then conducted a third literature search to identify technology use assessments or surveys, such as the Pew Internet and American Life Project that evaluated digital media and technology use [1]. Relevant items were added to the item pool.

Our second approach to develop a robust item pool involved seeking input from experts in the field. We convened an in-person meeting with 24 scientists across disciplines whose work related to digital technology. Their backgrounds encompassed the fields of psychology, social work, public health, statistics, economics, anthropology, communication, and medicine. During the convening, we presented the goal and process of the scale development project. We then provided a document with the 3 theoretical frameworks, (Uses and Gratifications, the FIM, and Affordances). We also listed all proposed items from our literature review on the document. Experts met in groups of 4 to 5 people for discussion; we asked for their written feedback on proposed items, as well as generation of new items to represent any proposed items that were missing.

All relevant items from both the literature review and expert convening were incorporated into the initial item pool. The initial item pool consisted of 71 items, of which 60 resulted from the literature search and 11 arose from the expert convening.

### Item Pool Refinement

To refine the initial item pool, we first removed any items that were duplicates. Second, we conducted an iterative process among an interdisciplinary team of investigators to discuss similar items. This process involved identifying items representing similar concepts but differed in scope. An example item would be *tagging friends* as a broader item and *tagging friends in a photo album* as a narrower item. These items were reviewed and discussed. We used a consensus approach to identify how to collapse similar items such as this into a single item. At this stage, we also discussed and proposed the item response scale. On the basis of similar scales in the literature, our goal was to use a Likert scale to capture variations along a response scale. Similar to many previous studies, we proposed a 5-item response scale from "extremely important" to "not at all important".

A final stage of item pool refinement involved pilot testing the item pool among a group of 8 adolescents aged 15 to 18 years. These reviews were conducted in a stepwise fashion of 1 to 2 adolescent interviews per step, with iterations of the item pool between each step. Through cognitive interviews, we asked for interpretations of each item, and feedback on items that were confusing. Items that were flagged as confusing were revised, items that were identified as uncommon or considered not relevant to adolescents were removed. We also asked adolescents to suggest any key concepts were missing from the item pool and should be represented. Finally, we asked adolescents for any feedback on the proposed Likert response scale. At the final step of interviews, no further revisions were suggested and thus we conclude this process. Our refined item pool consisted of 40 items.

### Data Collection

Data collection for item pool testing was conducted using a closed cross-sectional Web-based survey to reach a national sample of adolescents. Data were collected between November 2018 and January 2019. We used Qualtrics as our Web-based survey platform and for panel-based recruitment. Qualtrics recruits panelists with Web-based advertisements (eg, on social media or in mobile apps), inviting survey participation as a way to earn credit toward rewards, such as gift cards, in-app purchases, or airline miles. A background check is conducted to verify identity before the participant becomes part of a panel and eligible for recruitment. Surveys deployed via Qualtrics panels typically demonstrate demographic characteristics that fall within a 10% range of the values observed in the US population [30].

### Participants and Recruitment

The target population for this study was 12- to 18-year-olds who were US residents and English speaking. We established the parameters for Qualtrics to recruit a sample consistent with race/ethnicity representative of the US census population for 12- to 18-year-olds. Parameters for survey completion designated that any participants who completed less than half the survey were considered nonresponsive and data were excluded by Qualtrics before data delivery to investigators. Recruitment approaches were modeled after previous youth and media studies using Qualtrics [31].

A recruitment message was emailed to potentially eligible individuals notifying them of a survey opportunity, describing the estimated survey length (15 min), and informing them that e-rewards credit could be obtained in return for participation. All 18-year-old participants provided informed consent. Minor participants provided informed assent and their legally authorized guardians provided parental consent. All participants were instructed to complete the survey independently in a private location.

### Web-Based Survey

The survey comprised: (1) the refined item pool, (2) a short form of the Marlowe-Crown Social Desirability scale [32], and (3) demographic questions (Multimedia Appendix 1).

Participants were asked to rank each of the 40 items by importance. For each item, participants were asked “How important, if at all, is it for you to use media and technology platforms for the following purposes?” Participants responded using a 5-point Likert scale ranging from “not at all important” to “extremely important.”

The Marlowe-Crown Social Desirability scale was designed to identify participant responses that suggest a bias toward social desirability. This scale has 10 items, example items include “I am always willing to admit it when I make a mistake” and “I like to gossip.” Response options include true and false. High scores on this scale suggest answers may be biased by social desirability. This scale has been used in previous studies to evaluate items during the scale development process [33].

Demographic data included age, sex, race/ethnicity, and parental education. All items provided a nonresponse option, and participants were able to review and change answers before submitting.

### Analyses

Study data were delivered securely to investigators without participant identifiers. An initial review of survey data was conducted by investigators for 2 main types of data quality checks. First, we identified any participants who had completed the survey in <2 min. Qualtrics provides response time for every participant, and we calculated the average response time across the study population. We identified 2 min as our target cutoff as it represented less than 10% of the average response time. Second, we identified any participants who had responded with all responses using a single answer, for example, if all response options were the same multiple-choice option across all scales. We also reviewed any suspicious participant responses for *Christmas tree* patterns in which responses were present in a stepwise pattern throughout the survey (eg, multiple choice response patterns such as ABCDEDCBA). Data from these participants (n=36) were removed from our data set. Qualtrics then conducted recruitment for an additional 36 participants within original survey parameters.

Statistical analyses were performed using the MPlus software (Muthen and Muthen, version 8; California) to conduct exploratory factor analysis (EFA) and confirmatory factor analysis (CFA). All *P* values were 2-sided, and *P*<.05 was used to indicate statistical significance. Descriptive statistics were summarized as frequencies and percentages or means (SD). Participant data from the Web-based survey were randomly split into a development subsample (n=500) and a test subsample (n=261) [34].

#### Development Subsample: Exploratory Factor Analysis

Within the development subsample of 500 participants, an iterative EFA with Promax rotation was conducted to explore the scale’s factor structure and reduce the total number of items. The Kaiser-Guttman criterion was used as the primary tool for determining the number of factors retained. We reviewed each item in an iterative 4-step process with 2 biostatisticians and 2 investigators present. First, we removed items with low factor loadings (loading of less than 0.4) or multiple cross loadings (more than 2 factors with loadings within 0.1 of each other). Second, we reviewed all items for the theoretical contribution and factor loadings to ensure items were unique and represented distinct concepts within factors. Third, each item was assessed individually based on variation in responses and item-scale correlation. Items with item-scale correlation of less than 0.2 were removed. Fourth, the association between each item and social desirability scale scores was calculated using the Jackson Differential Reliability Index (DRI) [35]. Items with a DRI approaching zero are highly associated with social desirability. With this draft scale, we then used a scree plot to confirm the items across the selected number of factors. Cronbach alpha values were computed to determine the internal consistency of the instrument.

#### Test Subsample: Confirmatory Factor Analysis

Analyses were repeated in the test subsample of 261 participants using a CFA model. Model parameters were estimated using the maximum likelihood approach. The following fit indices were evaluated based on Hu and Bentler’s recommendations [36]: (1) maximum likelihood‐based standardized root mean-squared residual (SRMR, desired value 0.08 or less, indicating good fit); (2) comparative fit index (CFI, desired value 0.95 or greater); and (3) root mean square error of approximation (RMSEA, desired value 0.06 or less, acceptable value 0.08 or less) along with the corresponding 95% CI and chi‐square value.

## Results

### Participants

A total of 761 adolescents completed the Web-based survey. The sample was 52.8% (402/761) female, 77.5% (590/761) white, and the mean age was 14.8 (SD 1.7) years (Table 1).

**Table 1:** Participant characteristics (N=761).

Characteristics		Value, n (%)
**Gender**		
	Female	402 (52.8)
	Male	355 (46.6)
	Nonbinary gender	2 (0.3)
	Prefer not to answer	2 (0.3)
**Race**		
	White	590 (77.5)
	Black or African American	78 (10.3)
	Asian/Pacific Islander	64 (8.4)
	Hispanic/Latino	14 (1.8)
	American Indian/Hawaiian/Alaska Native	11 (1.5)
	Prefer not to answer	3 (0.4)
	Multiracial	1 (0.1)
**Highest grade completed**		
	5th	1 (0.1)
	6th	14 (1.8)
	7th	139 (18.3)
	8th	113 (14.8)
	9th	137 (18)
	10th	140 (18.4)
	11th	119 (15.6)
	12th	68 (8.9)
	Freshman in college	8 (1.1)
	Sophomore in college	12 (1.6)
	Other	6 (0.8)
	Prefer not to answer	4 (0.5)
**Parent education**		
	Less than high school	222 (29.2)
	High school or General Educational Development	168 (22.1)
	Some college or associate’s degree	171 (22.5)
	Bachelor’s degree	121 (15.9)
	Advanced degree (master’s, PhD, MD, etc)	72 (9.4)
	Prefer not to answer	7 (0.9)

### Development Subsample: Exploratory Factor Analysis

After removing items that did not meet criteria through our 4 assessments, there were 18 items remaining. The final model from the developmental subsample indicated 18 items remained in a 3-factor model, with Cronbach alpha values for the 3 factors of 0.87 (factor 1), 0.90 (factor 2), and 0.82 (factor 3). All factors had alphas above 0.8, which indicates excellent internal consistency.

### Test Subsample: Confirmatory Factor Analysis

**Figure 1:**
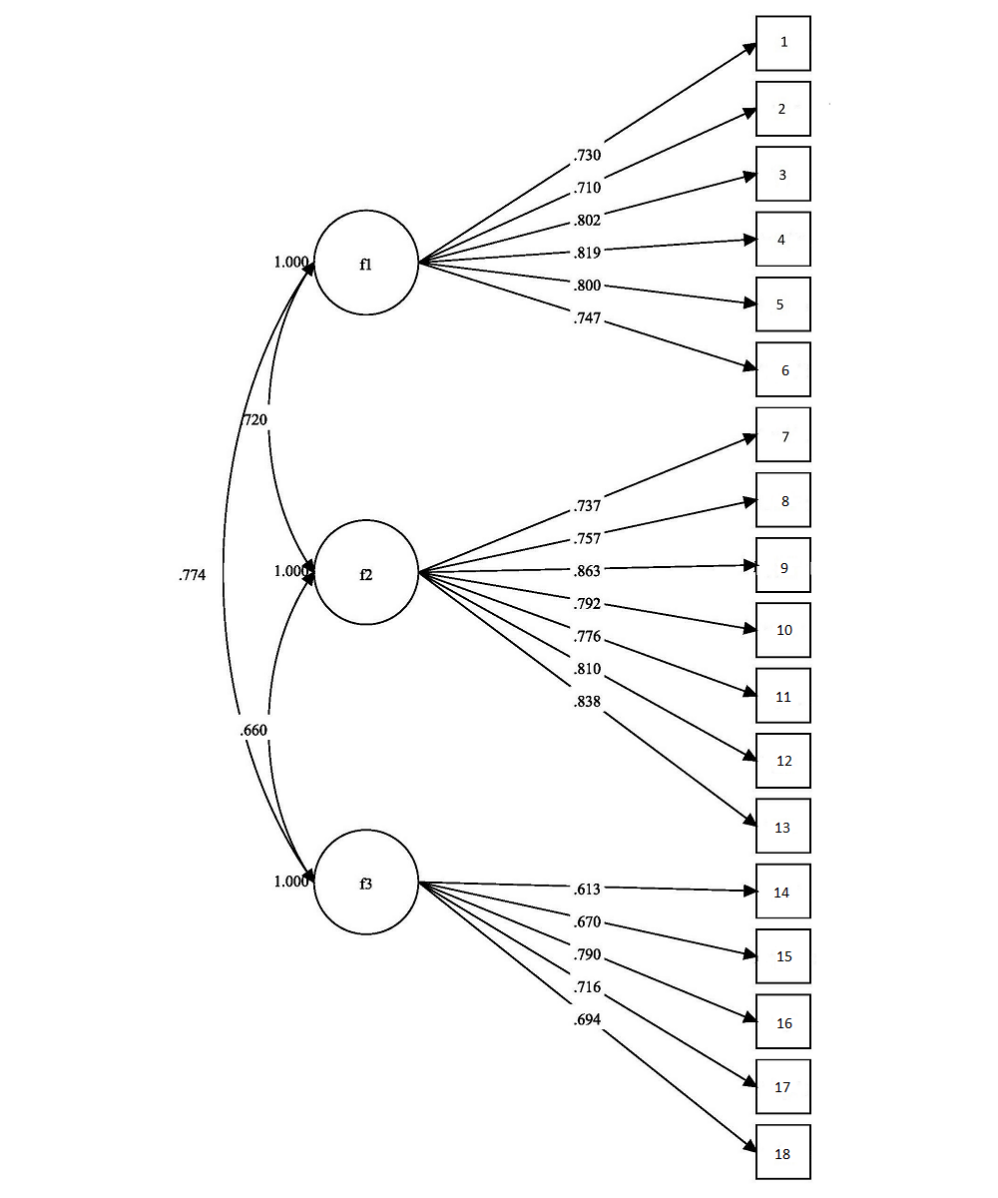
Factor structure with standardized loading for the 18-item Adolescents’ Digital Technology Interactions and Importance scale. f1: factor 1; f2: factor 2; f3: factor 3.

Scale fit indices included the following: the RMSEA was 0.063 (90% CI: 0.052-0.074), the CFI value was 0.952, and the SRMR value was 0.05. Across all measures, the values indicated good fit. The scale was finalized with 18 items. Figure 1 shows the factor structure and standardized factor loadings resulting from the CFA.

### Adolescents’ Digital Technology Interactions and Importance Scale

**Figure 2:**
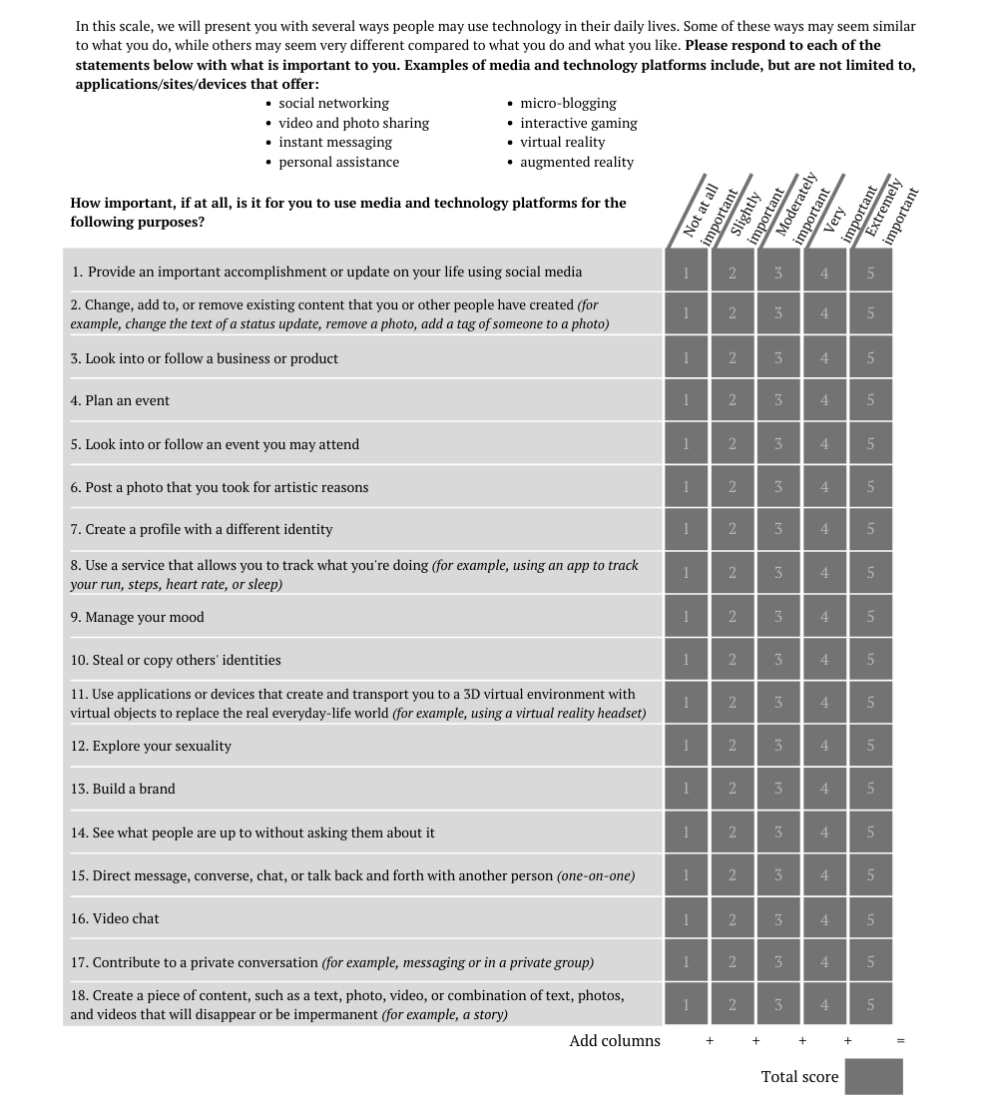
The Adolescents’ Digital Technology Interactions and Importance scale.

The scale was confirmed to have a 3-factor structure. Figure 2 shows the final version of the scale with response options.

#### Factor Structure

The first factor included items such as *provide an important accomplishment or update on your life using social media* and *follow or look into an event you may attend*. These items often represented sharing offline content about oneself online. These items also represented investigating offline people, businesses, or events in an Web-based space. Thus, this factor was labeled as *Technology to bridge online and offline experiences and preferences*.

Factor 2 included items such as *create a profile with a different identity*, *manage my mood,* and *use applications or devices that create or transport me to a virtual environment*. These items often represented ways for technology to assist an individual in going outside one’s current identity, mood, or offline environment. This factor was therefore named *Technology to go outside one’s identity or offline environment*.

Factor 3 included example items such as *videochat*, *see what people are up to without asking them about it*, and *contribute to a private* *conversation*. This factor was thus named *Technology for social connection*. Table 2 shows the descriptive data from each factor in the CFA sample.

**Table 2:** The Adolescents’ Digital Technology Interactions and Importance scale: descriptive information for 3-factor structure (n=261).

Factor number	Factor name	Value, mean (SD)^a^	Minimum value^b^	Maximum value^c^
1	Technology to bridge online and offline preferences and experiences	16.6 (6.4)	6	30
2	Technology to go outside one’s identity or offline environment	13.6 (7.5)	7	35
3	Technology for social connection	14.5 (5.1)	5	25

^a^Total=44.7 (SD 16.6).

^b^Total=18.

^c^Total=90.

## Discussion

### Principal Findings

This study contributes a new validated instrument for understanding how adolescents interact with and value interactive digital technologies. The Adolescents’ Digital Technology Interactions and Importance (ADTI) scale is grounded in theory, including the Uses and Gratifications model, the FIM, and the Affordances approach. Furthermore, the ADTI incorporates input from expert scientists as well as adolescents. The scale assesses the types of technology interactions rather than specific platforms, there are no *brand-name* platforms or programs included in the assessment items. Thus, the ADTI scale may be used over time as popular platforms emerge, peak, and decline. The ADTI also assesses interactions with novel technology, such as VR. The scale allows adolescents to report on technology interactions that are important to them, bypassing recall bias issues with reporting quantity of time. The focus on importance is unlikely to be subject to recall bias, as the adolescents are likely to report interactions that are most important to them at the time of taking the scale. Finally, the ADTI demonstrated strong psychometric validation through the EFA and CFA used in this study.

### Use of the Adolescents’ Digital Technology Interactions and Importance Scale

There are several ways in which the ADTI scale can be used in future research. First, the ADTI produces an overall score that represents a summary score of adolescents’ perceived importance of their interactions with technology. Thus, a high score indicates either moderate importance across many dimensions of technology or a focused importance on fewer items. A very high score may thus indicate adolescents who find extreme importance across many facets of technology use. Future studies to assess whether a particular high score as a cutoff is an indication of overemphasis on technology, or an overreliance on technology at the expense of offline experiences, may be warranted. However, the total score provides less nuance compared with the use of subscale scores.

The 3 factor subscales within the ADTI represent the distinct types of technology behaviors and interactions. These subscales have utility for future studies to understand whether certain subscale score ranges are associated with health or well-being outcomes. For example, higher levels of media use have been associated with loneliness [37]. Examining whether high or low scores on certain subscales, such as *technology for social connection*, are associated with loneliness may allow a more focused examination of this relationship.

It is also possible that the 3 factors in the ADTI scale could be used to understand ways that adolescents place value on their technology use as they navigate the developmental time period of adolescence. Adolescence is understood as a time for identity exploration, it is possible that *technology to go outside one’s identity or environment* is a stronger endorsed factor at certain times in adolescence [38]. Furthermore, investigators may opt to use selected questions or question groups to understand whether specific interactions are more important to certain groups of adolescents. For example, the items around exploring identity or sexuality may be more important to adolescents who identify as Lesbian, Gay, Bisexual, Transgender, Queer or Questioning, Intersex and use technology to explore or represent their identity [39]. Understanding common patterns in the importance of factors within the ADTI may assist in identifying technology use that is productive and healthy compared with that which is detrimental or risky.

### Limitations

This scale development study is not without limitations. Our item pool was generated from key theoretical approaches within the technology literature, it is possible that we overlooked less well known but important theories. We did note some overlap in the theoretical approaches we included. For example, social connection was featured across Uses and Gratifications, the FIM, and the Affordances approaches. Thus, the likelihood of ignoring a critical concept was less likely by drawing from several conceptual approaches. Furthermore, we consulted a group of interdisciplinary experts to ensure key concepts were not missed. Through our item reduction process, we eliminated items that did not have statistical support, it is possible that important concepts or items for some investigators or research disciplines were removed through this process. However, we relied upon validated processes to develop and test the ADTI scale, processes which are designed to create scales with high reliability and replicability. We involved adolescents in the item pool review process, which included reviewing items for understanding as well as relevance. We did ask adolescents for any concepts that were missing and needed to be added. However, our scale development process did not involve adolescent input at each stage of the project.

A limitation of this study is that our results may not generalize beyond a study population recruited via Qualtrics. Recruiting from a national panel of participants meant that we could achieve broad reach in recruitment but limited our ability to assess external validity of the sample. However, the Qualtrics platform and panels have been used in other studies of adolescents [31], and the panels have been found to have close approximations of US populations [30]. We did note a lower than expected Latino/Hispanic sample within our study population and plan to conduct additional studies to ensure the ADTI is tested in this group.

### Next Steps and Conclusions

Findings from our study, and those that we hope follow this line of work, will advance the scientific understanding and public dialogue on technology and adolescents. Previous work assessing consequences of technology use has nearly universally relied upon assessments of technology use time. Although time spent using technology remains an important measurement, it does not advance our understanding of the differential impact of how that individual chooses to prioritize their technology interactions.

There are several potential future directions for this scale. First, we plan to test the scale alongside existing measures of technology use to further assess convergent and divergent validity. We also plan to test the subscales alongside common health outcomes associated with technology use, including mental health outcomes such as depression, and wellness outcomes such as social support. Another potential future direction is that the ADTI scale could be included on future studies assessing technology and health or well-being outcomes. For example, items from the ADTI could be tested further for inclusion in large-scale studies such as the Youth Risk Behavior Survey [40] or the Pew Internet and American Life surveys [1]. Multimedia Appendix 2 includes the full scale and subscale items so that future studies can be conducted using the ADTI. In conclusion, the ADTI scale presents a promising new approach, informed by previous research and input from scientific experts, as well as adolescents themselves, to understand the value of teen technology use in their daily lives.

## Appendix

Multimedia Appendix 1 Scale development survey.

Multimedia Appendix 2 Adolescents’ Digital Technology Interactions and Importance scale and guidelines.

## Abbreviations

AAP: American Academy of Pediatrics
ADTI: Adolescents’ Digital Technology Interactions and Importance
CFA: confirmatory factor analysis
CFI: comparative fit index
DRI: differential reliability index
EFA: exploratory factor analysis
FIM: Facebook Influence Model
RMSEA: root mean square error of approximation
SRMR: standardized root mean-squared residual
VR: virtual reality
