# Supplementary material for: Measuring Interests Not Minutes: Development and Validation of the Adolescents’ Digital Technology Interactions and Importance Scale (ADTI)
Source: J Med Internet Res. 2020 Feb 12;22(2):e16736. doi: 10.2196/16736 (PMC7055832; doi:10.2196/16736)
Supplement: Multimedia Appendix 1 [file jmir_v22i2e16736_app1.pdf]

This is a Multimedia Appendix to a full manuscript published in the J Med Internet Res. For full copyright and citation information, see http://dx.doi.org/10.2196/16736

Online Questionnaire Distributed via Qualtrics:

Start of Block: Introduction Parent vs. Teen

Q1
You are invited to participate in a study with the purpose of understanding the role of media use by youth. Your answers may benefit the health of those in the future.

You will be instructed when the parent portion is complete and when the teen portion will begin if necessary. We appreciate you taking the time to participate.

Q2 Are you a parent or teen?

○ I am a parent (1)

○ I am a teen (2)

End of Block: Introduction Parent vs. Teen

Start of Block: Teen- Age No Parents

Q3 What is your age?

▼ 9 years of age or younger (1) ... 19 years of age or older (11)

Skip To: End of Block If What is your age? = 9 years of age or younger
Skip To: End of Block If What is your age? = 10 years of age
Skip To: End of Block If What is your age? = 11 years of age
Skip To: End of Block If What is your age? = 19 years of age or older

End of Block: Teen- Age No Parents

Start of Block: Teen Only Consent

Q4
Please ensure you are in a quiet place where you have privacy to complete the rest of this

survey. Please read the consent information in the attached document and then indicate your consent decision below.
Consent Form

○ I consent to be in this survey (1)

○ I DO NOT consent to be in this survey (2)

Skip To: End of Block If Please ensure you are in a quiet place where you have privacy to complete the rest of this surve... = I DO NOT consent to be in this survey

End of Block: Teen Only Consent

Start of Block: Teen Only Permission

Q5
This is a parental permission form. Please have your parent read the information in the attached document and then indicate whether they give consent for you to participate below.
Parent Permission Form

○ I consent for my child to participate in this survey (1)

○ I DO NOT consent for my child to participate in this survey (2)

Skip To: End of Block If This is a parental permission form. Please have your parent read the information in the attached... = I DO NOT consent for my child to participate in this survey

Q6 Thanks for answering our question! Please allow your child to take the rest of the survey.

End of Block: Teen Only Permission

Start of Block: Teen- Assent

Q7 At this point we'd like you to continue with the survey independently. Please ensure you are in a quiet place where you have privacy. Please read the assent information in the attached document and then indicate your consent decision below.
Assent Form

○ I consent to be in this survey (1)

○ I DO NOT consent to be in this survey (2)

Skip To: End of Block If At this point we'd like you to continue with the survey independently. Please ensure you are in... = I DO NOT consent to be in this survey

End of Block: Teen- Assent

Start of Block: Parent Age & W/

Q8 I have a child who is:

▼ 9 years of age or younger (1) ... 19 years of age or older (11)

Skip To: End of Block If I have a child who is: = 9 years of age or younger

Skip To: End of Block If I have a child who is: = 10 years of age

Skip To: End of Block If I have a child who is: = 11 years of age

Skip To: End of Block If I have a child who is: = 19 years of age or older

---

Q9 Is your teen currently with you?

○ Yes (4)

○ No (5)

Display This Question:

If Is your teen currently with you? = No

Q10
We ask that you return to this survey when your child is present.

Thank you for your interest.

Skip To: End of Block If We ask that you return to this survey when your child is present. Thank you for your interest.() Is Displayed

End of Block: Parent Age & W/

Start of Block: 18 Child Thanks

Q11 Thanks for answering our questions! Please allow your child to take the rest of the survey.

End of Block: 18 Child Thanks

Start of Block: Parental Permission

Q12
This is a parental permission form regarding your teens participation. Please read the information in the attached document and then indicate your consent decision for your child below.
Parent Permission Form

○ I consent for my child to participate in the survey (1)

○ I DO NOT consent for my child to participate in this study (2)

Skip To: End of Block If This is a parental permission form regarding your teen’s participation. Please read the informati... = I DO NOT consent for my child to participate in this study

Q13 Thanks for answering our questions! Please allow your child to take the rest of the survey.

End of Block: Parental Permission

Start of Block: Teen- Age

Q14 **TEEN**

What is your age?

▼ 9 years of age or younger (1) ... 19 years of age or older (11)

Skip To: End of Block If TEEN What is your age? = 9 years of age or younger
Skip To: End of Block If TEEN What is your age? = 10 years of age
Skip To: End of Block If TEEN What is your age? = 11 years of age
Skip To: End of Block If TEEN What is your age? = 19 years of age or older

End of Block: Teen- Age

Start of Block: Teen- Assent

Q15 At this point we'd like you to continue with the survey independently. Please ensure you are in a quiet place where you have privacy. Please read the assent information in the attached

document and then indicate your consent decision below. Assent Form

○ I consent to be in this survey (1)

○ I DO NOT consent to be in this survey (2)

Skip To: End of Block If At this point we'd like you to continue with the survey independently. Please ensure you are in... = I DO NOT consent to be in this survey

End of Block: Teen- Assent

Start of Block: Teen- Consent

Q16
Please ensure you are in a quiet place where you have privacy to complete the rest of this survey. Please read the consent information in the attached document and then indicate your consent decision below.
Consent Form

○ I consent to be in this survey (1)

○ I DO NOT consent to be in this survey (2)

Skip To: End of Block If Please ensure you are in a quiet place where you have privacy to complete the rest of this surve... = I DO NOT consent to be in this survey

End of Block: Teen- Consent

Start of Block: Interactions & Demographics Block 1

Q17 Are you of Hispanic, Latino or Spanish origin or descent?

- ○ No, not of Hispanic, Latino, or Spanish origin (1)
- ○ Yes, Mexican American, Chicano (2)
- ○ Yes, Puerto Rican (3)
- ○ Yes, Cuban (4)
- ○ Yes, another Hispanic, Latino or Spanish origin (5) ____
- ○ Prefer not to answer (6)

Q18 Which response best describes your gender?

- ○ Female (1)
- ○ Male (2)
- ○ Non-binary gender (3)
- ○ Female to male transgender (4)
- ○ Male to female transgender (5)
- ○ Other (6) ____
- ○ Prefer not to answer (7)

Q19 These questions will be related to your media use.

On average, how much time do you spend interacting with media each day?

- Less than an hour (1)
- About an hour (2)
- 1-2 hours (3)
- 3-4 hours (4)
- 5-6 hours (5)
- 7 or more hours (6)

Q20 In this part of the survey we will present to you several ways people may use technology in their daily lives. Some of these ways may seem similar to what you do, while others may seem very different compared to what you do and what you like. **Please respond to each of the statements below with what is important to you. Examples of media and technology platforms include, but are not limited to, applications/sites/devices that offer**:

- social networking
- video and photo sharing
- instant messaging
- personal assistance
- micro-blogging
- interactive gaming
- virtual reality
- augmented reality

End of Block: Interactions & Demographics Block 1

Start of Block: Interaction 1

Q21 **How important, if at all, is it for you to use media and technology platforms for the following purposes?**

	Not at all important (1)	Slightly important (2)	Moderately important (3)	Very important (4)	Extremely important (5)
Create a piece of content, such as a piece of text, a photo, a video, or a combination of text, photos and videos that is permanent (For example: post a status update on social media, share a photo, write a blog or a tweet) (1)	○	○	○	○	○

End of Block: Interaction 1

Start of Block: Interaction 2

Q22 **How important, if at all, is it for you to use media and technology platforms for the following purposes?**

	Not at all important (1)	Slightly important (2)	Moderately important (3)	Very important (4)	Extremely important (5)
Provide an important accomplishment or update on your life using social media (1)	○	○	○	○	○

End of Block: Interaction 2

Start of Block: Interaction 3

Q23 **How important, if at all, is it for you to use media and technology platforms for the following purposes?**

	Not at all important (1)	Slightly important (2)	Moderately important (3)	Very important (4)	Extremely important (5)
Stay in touch with people you wouldn't call or text (1)	○	○	○	○	○

End of Block: Interaction 3

Start of Block: Interaction 4

Q24 **How important, if at all, is it for you to use media and technology platforms for the following purposes?**

	Not at all important (1)	Slightly important (2)	Moderately important (3)	Very important (4)	Extremely important (5)
Change, add to, or remove from existing content that you or other people have created (for example, change the text of a status update, remove a photo, and add a tag of someone on a photo) (1)	○	○	○	○	○

End of Block: Interaction 4

Start of Block: Interaction 5

Q25 **How important, if at all, is it for you to use media and technology platforms for the following purposes?**

	Not at all important (1)	Slightly important (2)	Moderately important (3)	Very important (4)	Extremely important (5)
Look into or follow a business or product (1)	○	○	○	○	○

End of Block: Interaction 5

Start of Block: Interaction 6

Q26 **How important, if at all, is it for you to use media and technology platforms for the following purposes?**

	Not at all important (1)	Slightly important (2)	Moderately important (3)	Very important (4)	Extremely important (5)
Plan an event (1)	○	○	○	○	○

End of Block: Interaction 6

Start of Block: Interaction 7

Q27 **How important, if at all, is it for you to use media and technology platforms for the following purposes?**

	Not at all important (1)	Slightly important (2)	Moderately important (3)	Very important (4)	Extremely important (5)
Follow or look into an event you may attend (1)	○	○	○	○	○

End of Block: Interaction 7

Start of Block: Interaction 8

Q28 **How important, if at all, is it for you to use media and technology platforms for the following purposes?**

	Not at all important (1)	Slightly important (2)	Moderately important (3)	Very important (4)	Extremely important (5)
Look into someone or a group you recently met to see what they are like or if you would fit in (1)	○	○	○	○	○

End of Block: Interaction 8

Start of Block: Interaction 9

Q29 **How important, if at all, is it for you to use media and technology platforms for the following purposes?**

	Not at all important (1)	Slightly important (2)	Moderately important (3)	Very important (4)	Extremely important (5)
Post a photo (1)	○	○	○	○	○

End of Block: Interaction 9

Start of Block: Interaction 10

Q30 **How important, if at all, is it for you to use media and technology platforms for the following purposes?**

	Not at all important (1)	Slightly important (2)	Moderately important (3)	Very important (4)	Extremely important (5)
Post a photo that you took for artistic reasons (1)	○	○	○	○	○

End of Block: Interaction 10

Start of Block: Interaction 11

Q31 **How important, if at all, is it for you to use media and technology platforms for the following purposes?**

	Not at all important (1)	Slightly important (2)	Moderately important (3)	Very important (4)	Extremely important (5)
Explore new information or updates (For example: browse news, check the weather) (1)	○	○	○	○	○

End of Block: Interaction 11

Start of Block: Interaction 12

Q32 **How important, if at all, is it for you to use media and technology platforms for the following purposes?**

	Not at all important (1)	Slightly important (2)	Moderately important (3)	Very important (4)	Extremely important (5)
Posting content without it being associated with your name (1)	○	○	○	○	○

End of Block: Interaction 12

Start of Block: Interaction 13

Q33 **How important, if at all, is it for you to use media and technology platforms for the following purposes?**

	Not at all important (1)	Slightly important (2)	Moderately important (3)	Very important (4)	Extremely important (5)
Look at what is popular or trending (1)	○	○	○	○	○

End of Block: Interaction 13

Start of Block: Interaction 14

Q34 **How important, if at all, is it for you to use media and technology platforms for the following purposes?**

	Not at all important (1)	Slightly important (2)	Moderately important (3)	Very important (4)	Extremely important (5)
Create a profile with a different identity (1)	○	○	○	○	○

End of Block: Interaction 14

Start of Block: Interactions & Demographics Block 2

Q35 What would you consider your race?

- ○ White/Caucasian (1)
- ○ Black or African American (2)
- ○ American Indian or Alaska Native (3)
- ○ Asian (4)
- ○ Asian Indian (8)
- ○ Other Asian (9) ____
- ○ Native Hawaiian/ other Pacific Islander (5)
- ○ Multiracial (6)
- ○ Other (7) ____
- ○ Prefer not to answer (10)

Q36 What is the highest grade you have completed?

- ○ 7th grade (10)
- ○ 8th grade (1)
- ○ 9th grade (2)
- ○ 10th grade (3)
- ○ 11th grade (4)
- ○ 12th grade (5)
- ○ Freshman college (6)
- ○ Sophomore college (7)
- ○ Other (8) ____
- ○ Prefer not to answer (9)

Q37 Thanks for answering our questions so far, you're doing great! The following questions are the most important and in this part of the survey we will continue to present to you several ways people may use technology in their daily lives. Some of these ways may seem similar to what you do, while others may seem very different compared to what you do and what you like. **Please respond to each of the statements below with what is important to you. Examples of media and technology platforms include, but are not limited to, applications/sites/devices that offer:**

- social networking
- video and photo sharing
- instant messaging
- personal assistance
- micro-blogging
- interactive gaming
- virtual reality
- augmented reality

End of Block: Interactions & Demographics Block 2

Start of Block: Interaction 15

Q38 **How important, if at all, is it for you to use media and technology platforms for the following purposes?**

	Not at all important (1)	Slightly important (2)	Moderately important (3)	Very important (4)	Extremely important (5)
See what people are up to without asking them about it (1)	o	o	o	o	o

End of Block: Interaction 15

Start of Block: Interaction 16

Q39 **How important, if at all, is it for you to use media and technology platforms for the following purposes?**

	Not at all important (1)	Slightly important (2)	Moderately important (3)	Very important (4)	Extremely important (5)
See who is connected to whom on social media (1)	o	o	o	o	o

End of Block: Interaction 16

Start of Block: Interaction 17

Q40 **How important, if at all, is it for you to use media and technology platforms for the following purposes?**

	Not at all important (1)	Slightly important (2)	Moderately important (3)	Very important (4)	Extremely important (5)
Look through your own profile and reflect on how you see yourself and how others see you (For example: thinking about your past, your travels, your appearance) (1)	○	○	○	○	○

End of Block: Interaction 17

Start of Block: Interaction 18

Q41 **How important, if at all, is it for you to use media and technology platforms for the following purposes?**

	Not at all important (1)	Slightly important (2)	Moderately important (3)	Very important (4)	Extremely important (5)
Scroll through a timeline/feed (1)	○	○	○	○	○

End of Block: Interaction 18

Start of Block: Interaction 19

Q42 **How important, if at all, is it for you to use media and technology platforms for the following purposes?**

	Not at all important (1)	Slightly important (2)	Moderately important (3)	Very important (4)	Extremely important (5)
Read, listen or watch something for fun (For example: watch movies, music, shows, etc…) (1)	○	○	○	○	○

End of Block: Interaction 19

Start of Block: Interaction 20

Q43 **How important, if at all, is it for you to use media and technology platforms for the following purposes?**

	Not at all important (1)	Slightly important (2)	Moderately important (3)	Very important (4)	Extremely important (5)
Review, rate, or give feedback about something (For example: rating an online service, reviewing an online purchase) (1)	○	○	○	○	○

End of Block: Interaction 20

Start of Block: Interaction 21

Q44 **How important, if at all, is it for you to use media and technology platforms for the following purposes?**

	Not at all important (1)	Slightly important (2)	Moderately important (3)	Very important (4)	Extremely important (5)
Direct message, converse, chat, or talk back and forth with another person (one-on-one) (1)	○	○	○	○	○

End of Block: Interaction 21

Start of Block: Interaction 22

Q45 **How important, if at all, is it for you to use media and technology platforms for the following purposes?**

	Not at all important (1)	Slightly important (2)	Moderately important (3)	Very important (4)	Extremely important (5)
Video Chat (1)	○	○	○	○	○

End of Block: Interaction 22

Start of Block: Interaction 23

Q46 **How important, if at all, is it for you to use media and technology platforms for the following purposes?**

	Not at all important (1)	Slightly important (2)	Moderately important (3)	Very important (4)	Extremely important (5)
Contribute to a private conversation (for example, messaging or in a private group) (1)	◯	◯	◯	◯	◯

End of Block: Interaction 23

Start of Block: Interaction 24

Q47 **How important, if at all, is it for you to use media and technology platforms for the following purposes?**

	Not at all important (1)	Slightly important (2)	Moderately important (3)	Very important (4)	Extremely important (5)
Contribute to a public conversation (For example: on a Forum or a Page) (1)	◯	◯	◯	◯	◯

End of Block: Interaction 24

Start of Block: Interaction 25

Q48 **How important, if at all, is it for you to use media and technology platforms for the following purposes?**

	Not at all important (1)	Slightly important (2)	Moderately important (3)	Very important (4)	Extremely important (5)
Interact with a virtual assistant (1)	◯	◯	◯	◯	◯

End of Block: Interaction 25

Start of Block: Interaction 26

Q49 **How important, if at all, is it for you to use media and technology platforms for the following purposes?**

	Not at all important (1)	Slightly important (2)	Moderately important (3)	Very important (4)	Extremely important (5)
Ask a question/poll/ask for recommendations (1)	○	○	○	○	○

End of Block: Interaction 26

Start of Block: Interaction 27

Q50 **How important, if at all, is it for you to use media and technology platforms for the following purposes?**

	Not at all important (1)	Slightly important (2)	Moderately important (3)	Very important (4)	Extremely important (5)
Create a piece of content, such as a text, photo, video, or combination of text, photos, and videos that will disappear or be impermanent (for example, a story) (1)	○	○	○	○	○

End of Block: Interaction 27

Start of Block: Interaction 28

Q51 **How important, if at all, is it for you to use media and technology platforms for the following purposes?**

	Not at all important (1)	Slightly important (2)	Moderately important (3)	Very important (4)	Extremely important (5)
Check or change your privacy settings (1)	○	○	○	○	○

End of Block: Interaction 28

Start of Block: Interaction 29

Q52 **How important, if at all, is it for you to use media and technology platforms for the following purposes?**

	Not at all important (1)	Slightly important (2)	Moderately important (3)	Very important (4)	Extremely important (5)
Use a service that allows you to track what you're doing (for example, using an app to track your run, steps, heart rate, or sleep) (1)	○	○	○	○	○

End of Block: Interaction 29

Start of Block: Interaction 30

Q53 **How important, if at all, is it for you to use media and technology platforms for the following purposes?**

	Not at all important (1)	Slightly important (2)	Moderately important (3)	Very important (4)	Extremely important (5)
Manage your mood (1)	○	○	○	○	○

End of Block: Interaction 30

Start of Block: Interaction 31

Q54 **How important, if at all, is it for you to use media and technology platforms for the following purposes?**

	Not at all important (1)	Slightly important (2)	Moderately important (3)	Very important (4)	Extremely important (5)
Record events/experiences to remember them (1)	○	○	○	○	○

End of Block: Interaction 31

Start of Block: Interactions & Demographics Block 3

Q55 What type of school do you attend?

- ○ Public school: Middle or high school (1)
- ○ Private school: Middle or high school (2)
- ○ Home schooled (3)
- ○ Online school (4)
- ○ Public 4-year college (5)
- ○ Private 4-year college (6)
- ○ Community college (7)
- ○ Trade school (8)
- ○ Not currently in school (9)
- ○ Prefer not to answer (10)

Q56 What is the highest grade or level of school completed by either of your parents/guardians?

- ○ Less than high school (1)
- ○ High school or GED (2)
- ○ Some college or Associate’s degree (3)
- ○ Bachelor’s degree (4)
- ○ Advanced degree (Master’s, Ph.D., M.D., etc.) (5)
- ○ Prefer not to answer (6)

Q57 Thank you for answering our questions, you're doing great! **We know you are tired, but there are only a few more questions in this final part** of the survey where we will present to you several ways people may use technology in their daily lives. Some of these ways may seem

similar to what you do, while others may seem very different compared to what you do and what you like. **Please respond to each of the statements below with what is important to you. Examples of media and technology platforms include, but are not limited to, applications/sites/devices that offer:**

- social networking
- video and photo sharing
- instant messaging
- personal assistance
- micro-blogging
- interactive gaming
- virtual reality
- augmented reality

End of Block: Interactions & Demographics Block 3

Start of Block: Interaction 32

Q58 **How important, if at all, is it for you to use media and technology platforms for the following purposes?**

	Not at all important (1)	Slightly important (2)	Moderately important (3)	Very important (4)	Extremely important (5)
Play video games on a computer, console, phone or other device (1)	○	○	○	○	○

End of Block: Interaction 32

Start of Block: Interaction 33

Q59 **How important, if at all, is it for you to use media and technology platforms for the following purposes?**

	Not at all important (1)	Slightly important (2)	Moderately important (3)	Very important (4)	Extremely important (5)
Steal or copy others' identities (1)	○	○	○	○	○

End of Block: Interaction 33

Start of Block: Interaction 34

Q60 **How important, if at all, is it for you to use media and technology platforms for the following purposes?**

	Not at all important (1)	Slightly important (2)	Moderately important (3)	Very important (4)	Extremely important (5)
Use maps to find my or others location or for navigation (1)	○	○	○	○	○

End of Block: Interaction 34

Start of Block: Interaction 35

Q61 **How important, if at all, is it for you to use media and technology platforms for the following purposes?**

	Not at all important (1)	Slightly important (2)	Moderately important (3)	Very important (4)	Extremely important (5)
Use applications or devices that create and transport you to a 3D virtual environment with virtual objects to replace the real everyday-life world. (for example, using a virtual reality headset) (1)	○	○	○	○	○

End of Block: Interaction 35

Start of Block: Interaction 36

Q62 **How important, if at all, is it for you to use media and technology platforms for the following purposes?**

	Not at all important (1)	Slightly important (2)	Moderately important (3)	Very important (4)	Extremely important (5)
Explore your sexuality (1)	○	○	○	○	○

End of Block: Interaction 36

Start of Block: Interaction 37

Q63 **How important, if at all, is it for you to use media and technology platforms for the following purposes?**

	Not at all important (1)	Slightly important (2)	Moderately important (3)	Very important (4)	Extremely important (5)
Build a brand (1)	○	○	○	○	○

End of Block: Interaction 37

Start of Block: Interaction 38

Q64 **How important, if at all, is it for you to use media and technology platforms for the following purposes?**

	Not at all important (1)	Slightly important (2)	Moderately important (3)	Very important (4)	Extremely important (5)
Explore people you may be interested in romantically (1)	○	○	○	○	○

End of Block: Interaction 38

Start of Block: Interaction 39

Q65 **How important, if at all, is it for you to use media and technology platforms for the following purposes?**

	Not at all important (1)	Slightly important (2)	Moderately important (3)	Very important (4)	Extremely important (5)
Tag to engage others in conversation (1)	○	○	○	○	○

End of Block: Interaction 39

Start of Block: Interaction 40

Q66 **How important, if at all, is it for you to use media and technology platforms for the following purposes?**

	Not at all important (1)	Slightly important (2)	Moderately important (3)	Very important (4)	Extremely important (5)
Create appealing or stylish appearance (1)	○	○	○	○	○

End of Block: Interaction 40

Start of Block: Social Desirability Scale

Q67 Listed below are a number of statements concerning personal attitudes and traits. Read each item and decide whether the statement is *true* or *false* as it pertains to you personally.

	True (1)	False (2)
I like to gossip. (1)	o	o
There have been occasions when I took advantage of someone. (2)	o	o
I'm always willing to admit it when I make a mistake. (3)	o	o
I always try to practice what I preach. (4)	o	o
I sometimes try to get even rather than forgive and forget. (5)	o	o
At times I have really insisted on having things my own way. (6)	o	o
There have been occasions when I felt like smashing things. (7)	o	o
I never resent being asked to return a favor. (8)	o	o
I have never been irked when people expressed ideas very different from my own. (9)	o	o
I have never deliberately said something that hurt someone's feelings. (10)	o	o

End of Block: Social Desirability Scale

Start of Block: End of Survey

Q192

This is the end of the survey. Thank you for taking our survey!

If any of these survey questions have acted as an emotional trigger or made you concerned for your own health and well-being, please contact your healthcare provider. If the threat to your health is urgent, please call Crisis Line at 866-4CRISIS or contact your local ER.

Thank you for participating in our study. The information you have provided will go towards improving the understanding of youth and digital technology use. For more information on our research, visit our website at www.smahrtresearch.com.
Please submit your survey answers before exploring the links.

End of Block: End of Survey
