# Supplementary material for: Measuring Interests Not Minutes: Development and Validation of the Adolescents’ Digital Technology Interactions and Importance Scale (ADTI)
Source: J Med Internet Res. 2020 Feb 12;22(2):e16736. doi: 10.2196/16736 (PMC7055832; doi:10.2196/16736)
Supplement: Multimedia Appendix 2 [file jmir_v22i2e16736_app2.pdf]

Adolescents' Digital Technology Interactions and Importance (ADTI) Scale

In this scale, we will present you with several ways people may use technology in their daily lives. Some of these ways may seem similar to what you do, while others may seem very different compared to what you do and what you like. **Please respond to each of the statements below with what is important to you. Examples of media and technology platforms include, but are not limited to, applications/sites/devices that offer:**

- social networking
- video and photo sharing
- instant messaging
- personal assistance
- micro-blogging
- interactive gaming
- virtual reality
- augmented reality

**How important, if at all, is it for you to use media and technology platforms for the following purposes?**	Not at all important	Slightly important	Moderately important	Very important	Extremely important
1. Provide an important accomplishment or update on your life using social media	1	2	3	4	5
2. Change, add to, or remove existing content that you or other people have created *(for example, change the text of a status update, remove a photo, add a tag of someone to a photo)*	1	2	3	4	5
3. Look into or follow a business or product	1	2	3	4	5
4. Plan an event	1	2	3	4	5
5. Look into or follow an event you may attend	1	2	3	4	5
6. Post a photo that you took for artistic reasons	1	2	3	4	5
7. Create a profile with a different identity	1	2	3	4	5
8. Use a service that allows you to track what you're doing *(for example, using an app to track your run, steps, heart rate, or sleep)*	1	2	3	4	5
9. Manage your mood	1	2	3	4	5
10. Steal or copy others' identities	1	2	3	4	5
11. Use applications or devices that create and transport you to a 3D virtual environment with virtual objects to replace the real everyday-life world *(for example, using a virtual reality headset)*	1	2	3	4	5
12. Explore your sexuality	1	2	3	4	5
13. Build a brand	1	2	3	4	5
14. See what people are up to without asking them about it	1	2	3	4	5
15. Direct message, converse, chat, or talk back and forth with another person *(one-on-one)*	1	2	3	4	5
16. Video chat	1	2	3	4	5
17. Contribute to a private conversation *(for example, messaging or in a private group)*	1	2	3	4	5
18. Create a piece of content, such as a text, photo, video, or combination of text, photos, and videos that will disappear or be impermanent *(for example, a story)*	1	2	3	4	5
Add columns	+	+	+	+	=

Total score

Scale Description

The Adolescents' Digital Technology Interactions and Importance (ADTI) scale is a validated scale intended to evaluate adolescents' digital technology interactions and their perceived importance.

The ADTI has 18 items and 3 factors: (1) Technology to bridge online/offline experiences (items 1-6), which assesses how adolescents share offline content about themselves online, and investigate offline people, businesses, or events, using online tools; (2) Technology to go outside one's identity or offline environment (items 7-13), which assesses ways for technology to assist an individual in going beyond their current identity, mood, or offline environment, and (3) Technology for social connection (items 14-18), which assesses ways adolescents use technology to relate, interact, or converse with others.

Reference

1. Moreno MA, Binger K, Zhao Q, Eickhoff, JC. The Adolescents' Digital Technology Interactions and Importance (ADTI) Scale: Development and Validation. Journal of Medical Internet Research. 2019.

For more information regarding the ADTI, or if you would like to use the ADTI for research or clinical practice, please contact:

Megan A. Moreno, MD, MSEd, MPH
PI of the Social Media and Adolescent Health Research Team (SMAHRT)
Professor of Pediatrics and Vice Chair of Digital Health, University of Wisconsin-Madison
Email: moreno@wisc.edu; smahrt@pediatrics.wisc.edu
Website: www.smahrtresearch.com
